# Posttransplant Intramuscular Injection of PLX-R18 Mesenchymal-Like Adherent Stromal Cells Improves Human Hematopoietic Engraftment in A Murine Transplant Model

**DOI:** 10.3389/fmed.2018.00037

**Published:** 2018-02-22

**Authors:** Leland Metheny, Saada Eid, Karen Lingas, Racheli Ofir, Lena Pinzur, Howard Meyerson, Hillard M. Lazarus, Alex Y. Huang

**Affiliations:** ^1^Stem Cell Transplant Program, University Hospitals Cleveland Medical Center and Case Western Reserve University, Cleveland, OH, United States; ^2^Divsion of Pediatric Hematology-Oncology, Department of Pediatrics, Case Western Reserve University, Cleveland, OH, United States; ^3^Angie Fowler AYA Cancer Institute, UH Rainbow Babies & Children’s Hospital, Cleveland, OH, United States; ^4^Pluristem LTD, Haifa, Israel; ^5^Department of Pathology, Case Western Reserve University School of Medicine, Cleveland, OH, United States; ^6^University Hospitals Cleveland Medical Center, Cleveland, OH, United States

**Keywords:** cord blood stem cell transplantation, mesenchymal stromal cells, hematopoietic stem cell transplantation, engraftment, posttransplant complications

## Abstract

Late-term complications of hematopoietic cell transplantation (HCT) are numerous and include incomplete engraftment. One possible mechanism of incomplete engraftment after HCT is cytokine-mediated suppression or dysfunction of the bone marrow microenvironment. Mesenchymal stromal cells (MSCs) elaborate cytokines that nurture or stimulate the marrow microenvironment by several mechanisms. We hypothesize that the administration of exogenous MSCs may modulate the bone marrow milieu and improve peripheral blood count recovery in the setting of incomplete engraftment. In the current study, we demonstrated that posttransplant intramuscular administration of human placental derived mesenchymal-like adherent stromal cells [PLacental eXpanded (PLX)-R18] harvested from a three-dimensional *in vitro* culture system improved posttransplant engraftment of human immune compartment in an immune-deficient murine transplantation model. As measured by the percentage of CD45^+^ cell recovery, we observed improvement in the peripheral blood counts at weeks 6 (8.4 vs. 24.1%, *p* < 0.001) and 8 (7.3 vs. 13.1%, *p* < 0.05) and in the bone marrow at week 8 (28 vs. 40.0%, *p* < 0.01) in the PLX-R18 cohort. As measured by percentage of CD19^+^ cell recovery, there was improvement at weeks 6 (12.6 vs. 3.8%) and 8 (10.1 vs. 4.1%). These results suggest that PLX-R18 may have a therapeutic role in improving incomplete engraftment after HCT.

## Introduction

The late-term complications of hematopoietic cell transplantation (HCT) are numerous and include, but are not limited to, chronic graft-vs.-host disease (cGVHD), immunodeficiency, opporunistic infections, liver, pulmonary, and endocrine dysfunction, gonadal failure, and secondary malignancy (1, 2). Slow recovery of blood counts is also a well-recognized complication of transplantation, as well as incomplete engraftment, especially after umbilical cord blood (UCB) grafts and T-cell depleted or *in vitro* manipulated grafts (3). The incidence of long-term thrombocytopenia varies from 5 to 20% of HCT patients (4–10). Cytopenia may be related to transplant specific complications, such as cGVHD, inadequate cell dose of the graft, marrow dysfunction, immunosuppressive medications, and late chemotherapeutic effects (11, 12). Cytopenia after transplant is a predictor of non-relapse mortality (13–15). Possible mechanisms of cytopenia after HCT include cytokine-mediated suppression of megakaryopoiesis, erythropoiesis, or lymphopoiesis, and dysfunction of the bone marrow microenvironment (16, 17). Bone marrow stromal cells such as mesenchymal stromal cells (MSCs) elaborate cytokines that nurture or stimulate the marrow microenvironment by several mechanisms (18–20). MSCs can act as pericytes, wrapping around the endothelial cells of capillaries and venules and secrete bioactive products that contribute to tissue regeneration (21, 22). MSCs could also be selectively immune-suppressive and could affect the production of inhibitory cytokines (23, 24). Hence, it is possible that administration of MSCs in the setting of incomplete or delayed engraftment can modulate the milieu of the bone marrow microenvironment, through both direct interaction with hematopoietic stem cells (HSCs) and through secretion of cytokines, to improve blood counts posttransplant (25–27).

Mesenchymal stromal cells given at the time of HCT have been shown to improve engraftment, as well as have immunomodulatory effects in murine stem cell transplantation models (28, 29). MSCs can be found within multiple site, including adipose tissue and bone marrow (30, 31). The placenta is also an easily available source of cells from mesenchymal origin (32). PLacental eXpanded (PLX)-R18 (Pluristem Ltd., Haifa, Israel) is a human placental derived mesenchymal-like adherent stromal cells grown *in vitro* in a three-dimensional system. These cells secrete cytokines which contribute to hematopoietic reconstitution and differentiation, including IL-6, MCP-3, HGF, IL-8, FGF-7, GM-CSF, IL-10, and bFGF (33). Previous studies have demonstrated that intramuscular (IM) injections of PLX-R18 can mitigate mortality from acute radiation syndrome in murine models (33). PLX-R18 is being developed under the FDA’s animal rule for hematopoietic rescue from radiation syndrome. The effects of IM PLX-R18 appear to be related to transient secretion of pro-differentiation and pro-growth cytokines (33, 34).

Because there is no mouse model of delayed or incomplete engraftment, we proposed to administer PLX-R18 posttransplant in a well-established murine model of transplant utilizing sub-optimal doses of human CD34-selected UCB after radiation conditioning. Our hypothesis is that posttransplant IM administration of PLX-R18 will improve human hematopoietic engraftment, as measured by a quantitative improvement in human hematopoietic (CD45), B-cell (CD19), T-cell (CD3), megakaryocytic (CD41), and granulocyte (CD13, CD14) lineages in the peripheral blood and bone marrow.

## Materials and Methods

### Mice

Non-obese Diabetic–Severe Combined Immunodeficiency–IL2Rgammanull (NSG) mice were obtained from breeding pairs originally purchased from Jackson Laboratories (Bar Harbor, ME, USA). NSG mice were bred in a pathogen-free unit and maintained in sterile cages. Mice were handled and cared with strict adherence to guidelines as established by the Animal Resource Center and following study protocols as approved by the Institutional Animal Care and Use Committee at Case Western Reserve University School of Medicine (IACUC protocol 2015-0118).

### PLX-18

PLacental eXpanded-R18 cells were produced and supplied by Pluristem Therapeutics, Inc. (Haifa, Israel). The PLX-R18 cells are mesenchymal-like adherent stromal cells derived from full-term placentas following Cesarean section. The PLX-R18 production process is composed of two major steps of isolation and culturing of the adherent stromal cells. In the first stage, adherent stromal cells are isolated from the placenta and passaged under two-dimensional cell growth conditions. Cells are then concentrated and cryopreserved. This intermediate cell stock is later thawed, passaged, and subsequently seeded for further expansion in three-dimensional growth in a bioreactor on non-woven fiber-made carriers, from which cells are subsequently harvested and cryopreserved.

PLacental eXpanded-R18 cells have a spindle-like morphology and are characterized by a high expression of typical MSC markers, such as CD105, CD73 and CD29, and lack surface expression of CD45, CD34, CD14, CD19, and HLA-DR. In addition, PLX-R18 does not express CD31 (an endothelial marker) and GlyA (an erythrocyte cell marker) on their surface. PLX-R18 cells exhibit limited capacity to differentiate *in vitro* into osteocytes and adipocytes compared to bone marrow-derived MSCs.

The cells are harvested and cryopreserved in liquid nitrogen as an “off the shelf” allogeneic adult cell source product. Prior to their administration the cells were thawed washed and suspended in Plasmalyte A solution (Baxter, Deerfield, IL, USA).

### CD34^+^ Umbilical Cord Cell Isolation

Umbilical cord blood units were received from the Cleveland Cord Blood Center (Cleveland, OH, USA). Each unit was diluted 1:3 with phosphate buffered saline (PBS) + 0.5% Human Serum Albumin (HSA) and layered onto Ficoll Paque PLUS to isolate the mononuclear cells by density gradient. After a cell count and washes, the mononuclear cells were labeled per protocol using the Miltenyi Biotec (Cologne, Germany) CD34 Microbead Kit. The CD34 cells were then isolated using a positive selection column in a magnet and washed three times with MACS buffer. The CD34 cells from both UCB units were combined, counted, and re-suspended in PBS for injection into mice.

### Murine Transplantation

**Figure 1:**
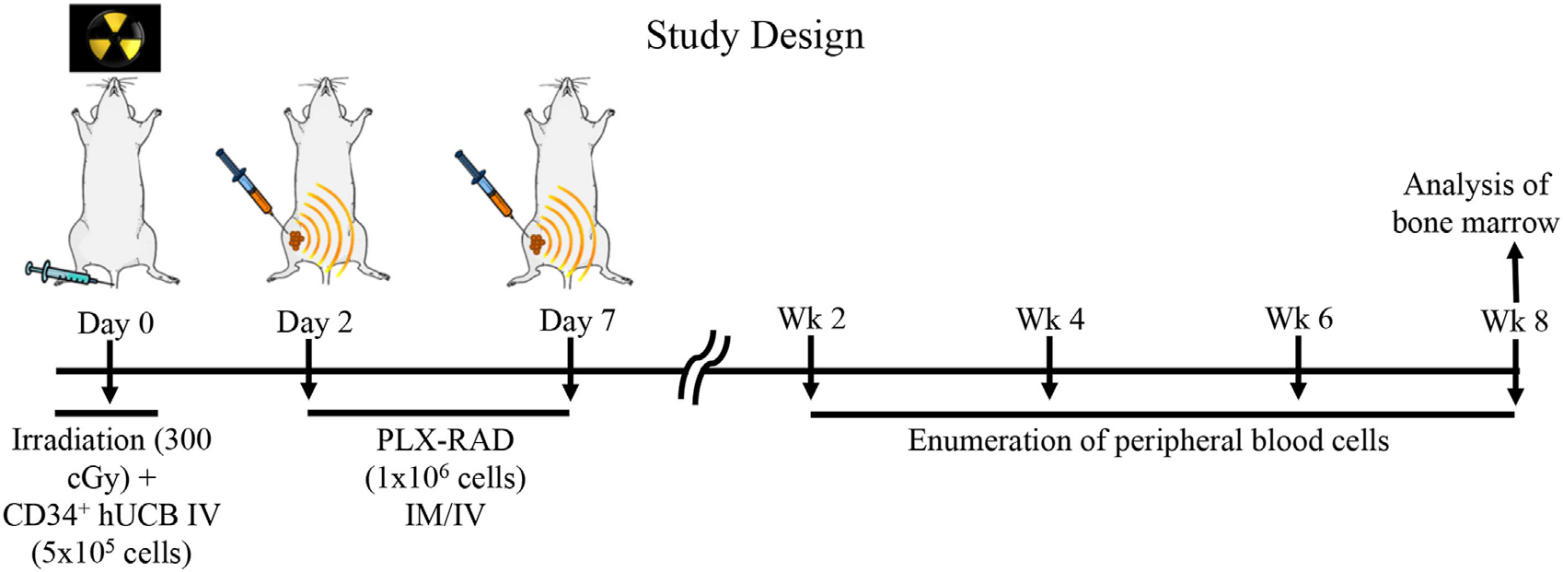
Study design: NSG mice received 300 cGy total body irradiation prior to receiving 5 × 10^5^ CD34^+^ selected cells from human umbilical cord blood (UCB). Following non-lethal irradiation (300 cGy), two groups of NSG mice were studied: (1) intravenous (IV) 5 × 10^5^ UCB CD34^+^ cells and (2) IV 5 × 10^5^ UBC CD34^+^ cells and 1 × 10^6^ intramuscular (IM) PLacental eXpanded (PLX)-R18 on D2 and D7. Peripheral blood from transplant recipient mice were analyzed on weeks 2, 4, 6, and 8. Bone marrow from transplant recipient mice was analyzed on week 8.

NSG mice aged 8–12 weeks were used in all transplant experiments. Prior to transplantation with UCB, recipient mice were ear punched for individual identification. NSG mice received 300 cGy total body irradiation prior to receiving 5 × 10^5^ CD34^+^ selected cells from human UCB, which has been shown to lead to incomplete human hematopoietic recovery (35). Following non-lethal irradiation (300 cGy), two groups of NSG mice were studied: (1) intravenous (IV) 5 × 10^5^ UCB CD34^+^ cells (*N* = 10) and (2) IV 5 × 10^5^ UBC CD34^+^ cells and 1 × 10^6^ IM PLX-R18 on day 2 (D2) and day 7 (D7) (*N* = 14) (Figure 1). Initially, IV and IM injection of PLX-R18 were attempted; however, mice immediately developed fatal acute pulmonary toxicity after IV infusion (even with slow infusion) and thus the IM route obviously was preferred.

### Injections

Umbilical cord blood cells were administered *via* tail vein injection suspended in a total volume of 200 µL. IM injections: PLX-18 cells were administered *via* IM right thigh injections suspended in a total volume of 100 µL.

### Tissue Harvest and Preparation

The liver, ileum, ascending colon, and right tibia were harvested and were fixed in 10% buffered formalin, embedded in paraffin, cut into 5-μm thick sections and stained with hematoxylin and eosin for histologic examination. Slides were coded without reference to transplant group or treatment and reviewed in blinded fashion by an independent pathologist (HM).

### Flow Cytometry

Peripheral blood samples from transplant recipient mice were analyzed on weeks 2, 4, 6, and 8. Bone marrow from transplant recipient mice was analyzed on week 8. Peripheral blood and bone marrow were stained for T cell markers (CD3), myeloid markers (CD45, CD13, CD14, CD41) along with B-cell markers (CD19). All monoclonal antibodies (mAbs) are human specific and were purchased from BD Biosciences Pharmingen (San Diego, CA, USA) or eBioscience (San Diego, CA, USA). At least 1 × 10^5^ events were analyzed per conjugated MAb stain condition. Data were analyzed using CFlow software (Accuri, Ann Arbor, MI, USA). Human engraftment was expressed as percentage of CD45^+^ cells within the gated population of bone marrow or peripheral blood cells. Engraftment of CD45^+^ cell subsets (CD3, CD13, CD14, CD19) was expressed as a percentage within the gated population of bone marrow or peripheral blood cells. Platelet engraftment was expressed as a percentage of CD41^+^ events compared to total peripheral blood or bone marrow cells.

### Histology

An independent hematopathologist, blinded to cohort characteristics, evaluated all tibial sections. The analysis included sections from each mouse in every cohort and was evaluated for myeloid-to-erythroid ratio, cellularity, and megakaryocyte percentages.

### Statistics

All values are expressed as the mean ± SEM. Statistical comparisons between groups were completed using Mann and Whitney test (nonparametric data).

## Results

### Posttransplant IM Administration of PLX-R18 Improves Overall Human Hematopoietic Cell Engraftment (CD45) and B-Cell Engraftment (CD19) in the Peripheral Blood

**Figure 2:**
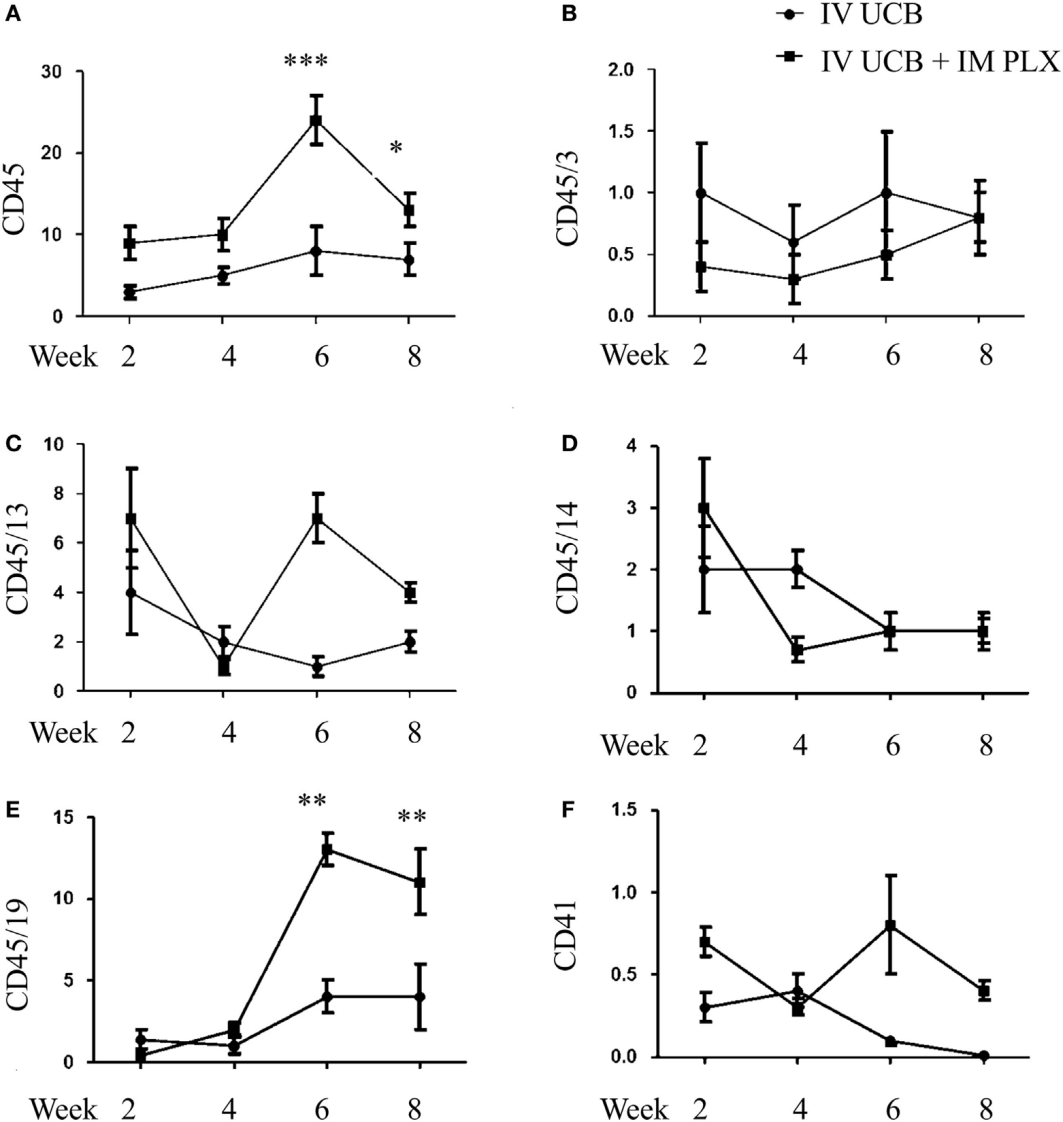
Posttransplant PLacental eXpanded (PLX)-R18 IM injection improves human leukocyte (CD45) and B-cell (CD45/19) engraftment in the peripheral blood. Flow cytometry results of CD45 **(A)**, CD45/CD3 **(B)**, CD45/CD13 **(C)**, CD45/CD14 **(D)**, CD45/CD19 **(E)**, and CD41 **(F)** in the peripheral blood at weeks 2, 4, 6, and 8 weeks are shown. IV UCB, *n* = 9; IV UCB/IM PLX, *n* = 14; IV, intravenous; UCB, umbilical cord blood; IM, intramuscular; *p < 0.05; **p < 0.01; ***p < 0.001.

**Figure 3:**
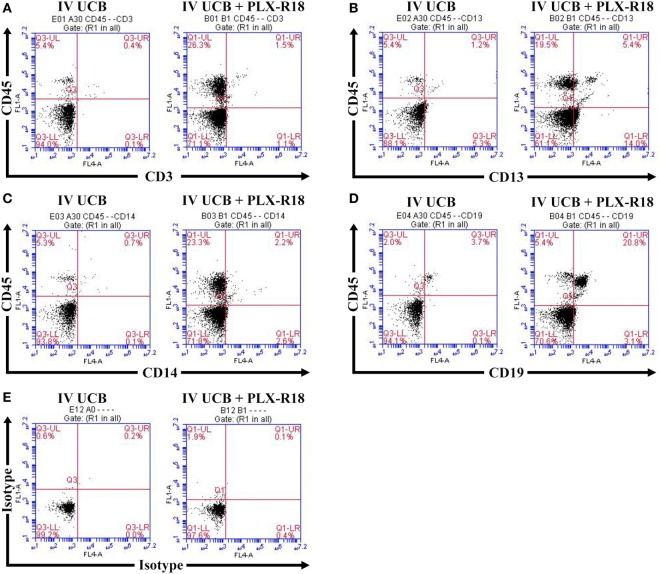
Representative flow cytometry plot of peripheral blood at 8 weeks posttransplant. CD45/CD3 **(A)** CD45/CD13 **(B)**, CD45/CD14 **(C)**, CD45/CD19 **(D)**, and isotype control **(E)**.

To create a mouse model of incomplete engraftment, we injected sub-optimal dose (1 × 10^5^ cells/mouse) of human UCB-derived CD34^+^ cells into NSG mice following 300 cGy whole-body irradiation. IM of 1 × 106 PLX-R18 were administered on days 2 and 7 following hUCB transplantation (Figure 1). Hematopoietic reconstitution analyses were performed every 2 weeks starting 2 weeks following hUCB transplantation. Bi-weekly flow cytometry analysis of the peripheral blood revealed the highest level of human CD45^+^ cells and B-cell engraftment in the IM PLX-R18 cohort at weeks 6 and 8, but not at weeks 2 and 4 (Figure 2). Specifically, there was a gradual increase in human CD45 percentage in the IV UCB cohort from week to week, the percentage of human CD45 engraftment in the peripheral blood spiked at week 6 in the IM PLX-R18 cohort, which was statistically significant (8.4 vs. 24.1%, *p* < 0.001; Figure 2A; Table 1). While a relative decrease in the percentage of human CD45 cells occurred in the peripheral blood of those mice treated with PLX-R18 at week 8 when compared to week 6, it was still superior when compared to the IV hUCB cohort (7.3 vs. 13.1%, *p* < 0.05, Figure 2A; Table 1). In contrast, mice treated with PLX-R18 demonstrated sustained B-cell engraftment superior to the control cohort at weeks 6 (3.8 vs. 12.6%, *p* < 0.01, Figure 2B; Table 1) and 8 posttransplant (4.1 vs. 10.1%, *p* < 0.01; Figure 2E; Table 1). Similar rises in the percentage of human T-cell, granulocyte, and platelet engraftment occurred in the peripheral blood of the PLX-R18 cohort, but these measurements were not statistically different from the IV hUCB cohort (Figures 2B–D,F; Table 1). Representative flow cytometry plots of peripheral blood analysis at 8 weeks posttransplant are shown in Figure 3.

**Table 1:** Summary of the percent CD45, CD45/3, CD45/13, CD45/14, CD45/19, and CD41 in the peripheral blood at 2, 4, 6, and 8 weeks post umbilical cord blood (UCB) infusion.

	Week 2 (%)	Week 4 (%)	Week 6 (%)	Week 8 (%)
CD45 intravenous (IV) UCB	3.2	5.4	8.4	7.4
CD45 IV UCB + intramuscular (IM) PLacental eXpanded (PLX)	8.7 (*p* > 0.05)	10.4 (*p* > 0.05)	24.1 (*p* < 0.001)	13.1 (*p* < 0.05)
CD45/3 IV UCB	1.1	0.6	0.1	0.8
CD45/3 IV UCB + IM PLX	0.4 (*p* > 0.05)	0.3 (*p* > 0.05)	0.5 (*p* > 0.05)	0.8 (*p* > 0.05)
CD45/13 IV UCB	4.4	1.8	1.3	1.5
CD45/13 IV UCB + IM PLX	6.7 (*p* > 0.05)	1.2 (*p* > 0.05)	7.4 (*p* > 0.05)	3.6 (*p* > 0.05)
CD45/14 IV UCB	2.2	1.8	1.0	1.0
CD45/14 IV UCB + IM PLX	3.1 (*p* > 0.05)	0.8 (*p* > 0.05)	1.4 (*p* > 0.05)	1.6 (*p* > 0.05)
CD45/19 IV UCB	1.4	1	3.8	4.1
CD45/19 IV UCB + IM PLX	0.4 (*p* > 0.05)	1.7 (*p* > 0.05)	12.6 (*p* < 0.01)	10.1 (*p* < 0.01)
CD41 IV UCB	0.2	0.4	0.1	0
CD41 IV UCB + IM PLX	0.7 (*p* > 0.05)	0.3 (*p* > 0.05)	0.7 (*p* > 0.05)	0.4 (*p* > 0.05)

*p-Values as compared to the intravenous UCB group*.

### Posttransplant IM Administration of PLX-R18 Improves Overall Human CD45 Lineage Cell Engraftment in the Bone Marrow

**Figure 4:**
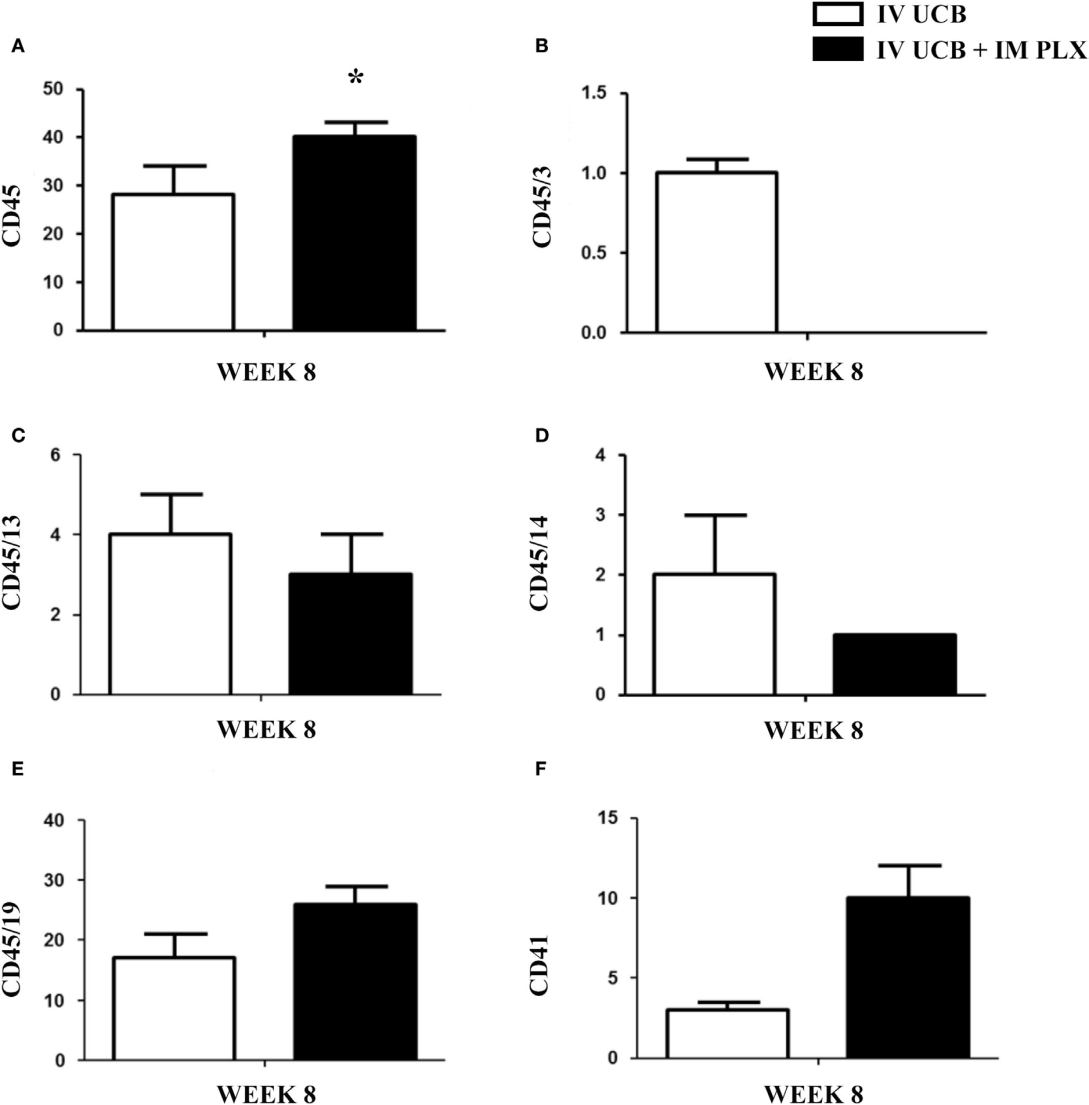
Posttransplant PLacental eXpanded (PLX)-R18 IM injection improves human leukocyte (CD45) in the bone marrow at 8 weeks. Flow cytometry results of the right tibia bone marrow CD45 **(A)**, CD45/CD3 **(B)**, CD45/CD13**(C)**, CD45/CD14 **(D)**, CD45/19 **(F)**, and CD41 **(E)**. IV UCB, *n* = 10; IV UCB/IM PLX, *n* = 14. IV, intravenous; UCB, umbilical cord blood; IM, intramuscular; *p < 0.05.

**Figure 5:**
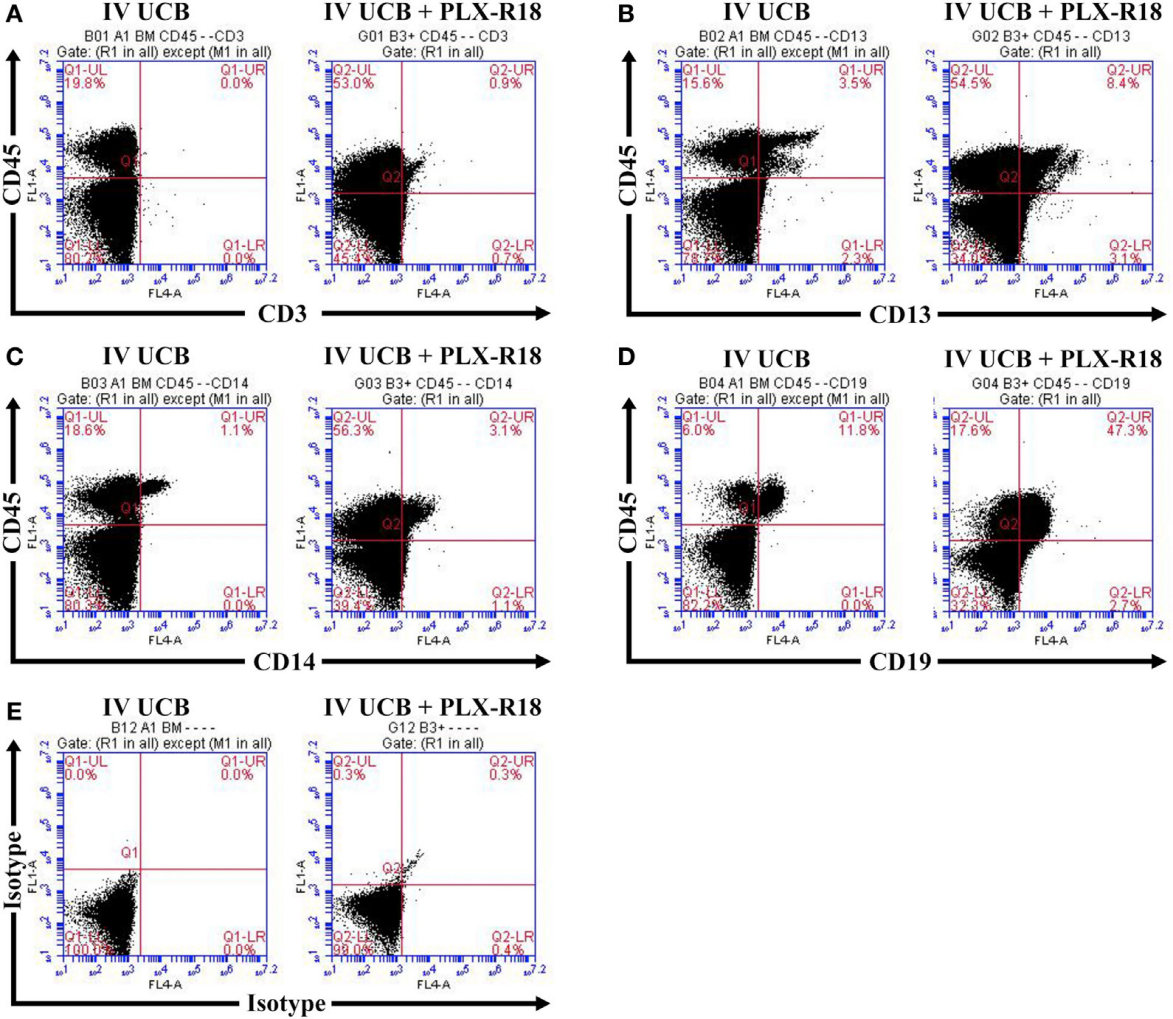
Representative flow cytometry plots of bone marrow at 8 weeks posttransplant. CD45/CD3 **(A)** CD45/CD13 **(B)**, CD45/CD14 **(C)**, CD45/CD19 **(D)**, and isotype control **(E)**.

**Figure 6:**
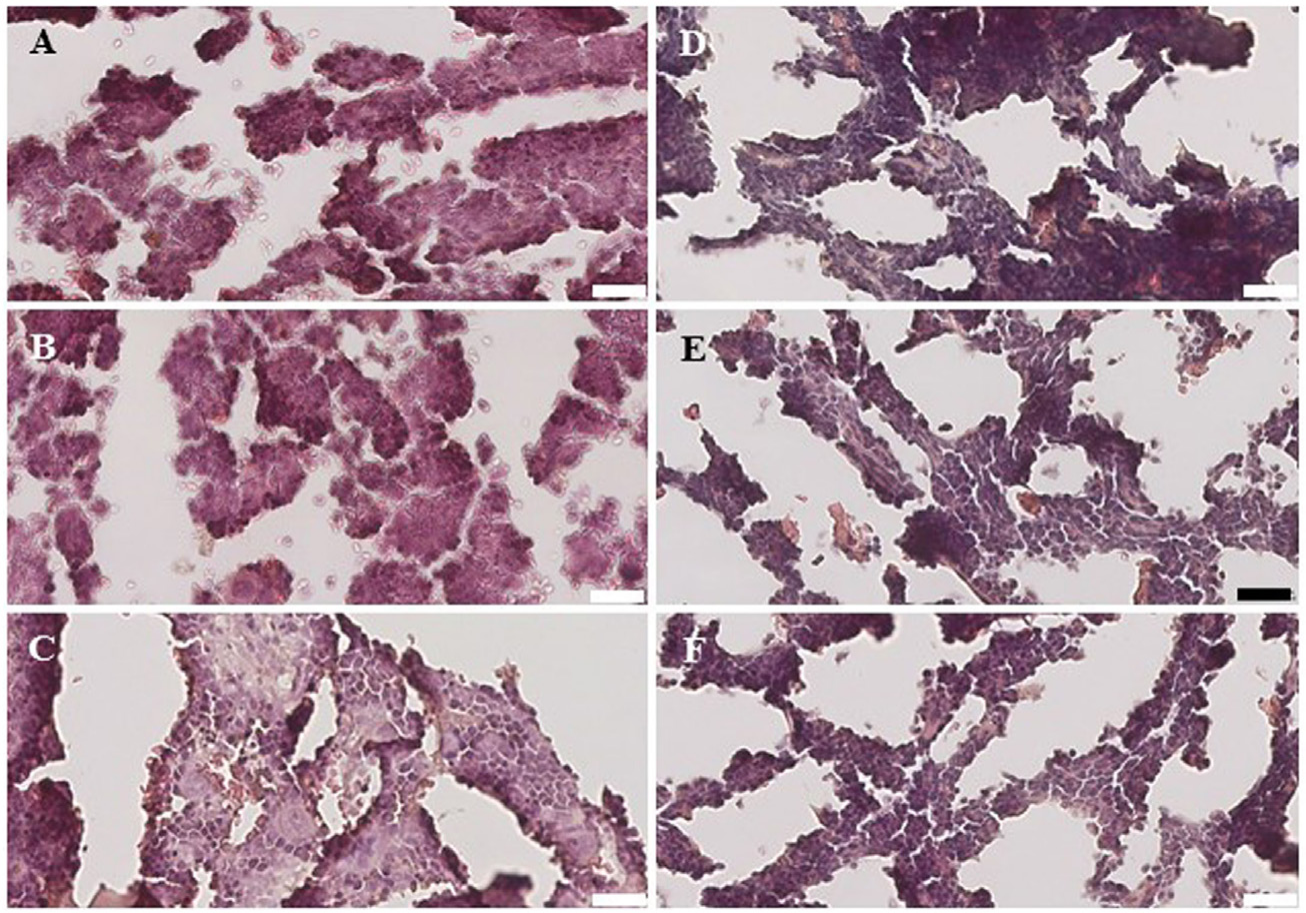
Histology of the bone marrow from NSG mice treated with intravenous (IV) 5 × 10^5^ umbilical cord blood CD34^+^ cells **(A–C)** and NSG mice treated with IV 5 × 10^5^ UBC CD34^+^ cells and 1 × 10^6^ intramuscular PLacental eXpanded-R18 on D2 and D7 **(D–F)**.

Within the bone marrow, the IV UCB/IM PLX-R18 cohort demonstrated similar increases in CD45^+^ lineage cells at 8 weeks as compared with the IV hUCB cohort control (28 vs. 40.0%, *p* < 0.01; Figure 4A; Table 2). In contrast with the peripheral blood, there was no significant difference in B-cells within the bone marrow between cohorts (Figure 4E); however, the majority of the CD45^+^ cells were also CD19^+^. The differences between megakaryocytes and platelets were not significance (2.6 vs. 10.2%, *p* > 0.05; Figure 4F; Table 2), nor were the differences between T-cells, granulocytes, and platelets or megakaryocytes in the bone marrow between either cohorts (Figures 4B–D; Table 2). Representative flow cytometry plots of bone marrow at 8 weeks posttransplant are shown in Figure 5. Concurrent histopathologic evaluation demonstrated no difference in overall cellularity, myeloid-to-erythroid ratio, or megakaryocytic percentages between the bone marrow samples of the cohorts at 8 weeks posttransplant (Figure 6).

**Table 2:** Summary of the percent CD45, CD45/3, CD45/13, CD45/14, CD45/19, and CD41 in the bilateral tibial and femoral bone marrow at 8 weeks post umbilical cord blood (UCB) infusion.

	CD45 (%)	CD45/3 (%)	CD45/13 (%)	CD45/14 (%)	CD45/19 (%)	CD41 (%)
IV UCB	28.0	1.3	3.7	2.1	17.4	2.6
IV UCB + intramuscular PLacental eXpanded	40.0 (*p* < 0.05)	0 (*p* > 0.05)	3.4 (*p* > 0.05)	1.1 (*p* > 0.05)	26.2 (*p* > 0.05)	10.2 (*p* > 0.05)

*p-Values as compared to the intravenous (IV) umbilical cord blood group*.

## Conclusion

Our results support the hypothesis that IM injection of PLX-R18 improves the rapidity of human UCB engraftment in NSG mice when given in the posttransplant setting. It is likely that PLX-R18 exert their effect through secretion of pro-differentiation and proliferative cytokines that subsequently exert systemic effects, given that the PLX-R18 cells were injected intramuscularly and not intravenously or intra-osseously (36). As such, the PLX-R18 cells are unlikely to track into the bone marrow microenvironment. Additional bio-distribution studies of IM PLX-R18 demonstrate that the cells remain local, but few survive beyond 13 weeks past administration (in press).

Consistent with previous experiments by Prather et al., the effect of the PLX-R18 appeared to be transient, as the percentage of CD45^+^ cells declined within the peripheral blood after week 6. This drop off after week 6 likely is the result of exhaustion, death, or clearance of the PLX-R18 cells (34). Interestingly, though the effect within the peripheral blood appeared transient, the week 8 analysis of the bone marrow did show a significant increase in human engraftment as expressed by CD45^+^ cells, suggesting that marrow engraftment can be enhanced and may be permanent. Cytokine analyses of the serum are ongoing, but *in vivo* experiments demonstrate that PLX-R18 secretes granulocyte-colony stimulating factor, CXCL1, monocyte chemo-attractant protein-1, and interleukin-6; these concentrations peak in the serum at day 9 post-IM injection (in press).

Intravenous instead of IM injection of placental derived MSC combined with hUCB seems to result in similar engraftment outcomes. In an NOD/SCID mouse model of transplant utilizing sub-lethal conditioning, placental derived MSCs grown in a 3D-culture (PLX-I) combined with hUCB resulted in improved engraftment rates in the bone marrow at 6 weeks as measured by CD45^+^ human cells (37). Experiments involving IV cotransplant of hUCB and MSC has demonstrated improved engraftment rates of CD45^+^ human cells in the peripheral blood and bone marrow of NOD/SCID mice at 6–8 weeks posttransplant (20, 38). Similar to IM PLX-R18 cohort, the majority of the CD45^+^ population of cells in both the bone marrow and the peripheral blood were of the lymphoid lineage when hUCB was co-transplant with MSC. This finding is also consistent with other studies and suggests that PLX-R18 may also improve immune reconstitution following transplant (28, 39).

A detailed histological analysis of the bone marrow did not demonstrate any differences in overall cellularity, megakaryocytic:erythrocytic (M:E) ratio, or the percentages of megakaryocytes among cohorts. In the setting of non-myeloablative radiation, autologous recovery of murine bone marrow cells along with hUCB engraftment would be expected, and PLX-R18 cells may have enhanced the rate of this recovery.

The current therapeutic strategy is intriguing in that it may improve engraftment in patients who have incomplete engraftment after HCT. There is significant evidence in mice that MSC improve hUCB hematopoietic cell engraftment (18, 19). In theory, the observed experimental benefit could be extended to patients whose marrows have experienced delayed or incomplete engraftment. The safety and potential efficacy of IV administration of MSCs has been demonstrated in humans (25, 26). In autologous transplants, autologous MSCs co-infused with HSCs leads to rapid recovery of blood counts (26). In allogeneic transplantation, Lazarus et al. published a study in which 46 matched related donor transplant patients were infused with matched related culture-expanded MSCs prior to the hematopoietic graft. There were no toxicities associated with infusion of the MSC, and engraftment of neutrophils and platelets occurred at a median time of 14 and 20 days, respectively. In contrast with IV administration of MSC, IM administration of mesenchymal-like PLX cells has been documented to improve hematopoietic recovery in a three-patient cohort study (40). As such, a translational, a phase I international trial is now underway [ClinicalTrials.gov number NCT03002519].

## Ethics Statement

Non-obese Diabetic–Severe Combined Immunodeficiency–IL2Rgammanull (NSG) mice were obtained from breeding pairs originally purchased from Jackson Laboratories (Bar Harbor, ME, USA). NSG mice were bred in a pathogen-free unit and maintained in sterile cages. Mice were handled and cared with strict adherence to guidelines as established by the Animal Resource Center and following study protocols as approved by the Institutional Animal Care and Use Committee at Case Western Reserve University School of Medicine (IACUC protocol 2015-0118).

## Author Contributions

Concept and design (LM, HL, and AH), collection and/or assembly of data (LM, SE, KL, and HM), data analysis and interpretation (LM, HM, HL, and AH), manuscript writing (LM, RO, LP, HL, and AH), and final approval of manuscript (LM, HL, and AH).

## Conflict of Interest Statement

The authors declare that the research was conducted in the absence of any commercial or financial relationships that could be construed as a potential conflict of interest. The reviewer KCA and the handling editor declared their shared affiliation.

## Abbreviations

MSCs, human mesenchymal stromal cells; HSC, Hematopoietic stem cells; UCB, Umbilical cord blood; IV, Intravenous; IM, Intramuscular; NSG, non-obese diabetic severe combined immunodeficiency- IL2Rgammanull; HCT, Hematopoietic cell transplant; mAbs, Monoclonal antibodies; PBS, Phosphate Buffered Saline; HSA, Human Serum Albumin; IACUC, Institutional Animal Care and Use Committee.

## References

[1] Copelan EA Hematopoietic stem-cell transplantation. N Engl J Med (2006) 354(17):1813–26. 10.1056/NEJMra052638 16641398

[2] Passweg JR Baldomero H Peters C Gaspar HB Cesaro S Dreger P Hematopoietic SCT in Europe: data and trends in 2012 with special consideration of pediatric transplantation. Bone Marrow Transplant (2014) 49(6):744–50. 10.1038/bmt.2014.55 24637898 PMC4051369

[3] Norkin M Lazarus HM Wingard JR. Umbilical cord blood graft enhancement strategies: has the time come to move these into the clinic? Bone Marrow Transplant (2013) 48(7):884–9. 10.1038/bmt.2012.163 22941377

[4] Bruno B Gooley T Sullivan KM Davis C Bensinger WI Storb R Secondary failure of platelet recovery after hematopoietic stem cell transplantation. Biol Blood Marrow Transplant (2001) 7(3):154–62. 10.1053/bbmt.2001.v7.pm11302549 11302549

[5] Bentley SA Brecher ME Powell E Serody JS Wiley JM Shea TC. Long-term engraftment failure after marrow ablation and autologous hematopoietic reconstitution: differences between peripheral blood stem cell and bone marrow recipients. Bone Marrow Transplant (1997) 19(6):557–63. 10.1038/sj.bmt.1700717 9085735

[6] Cassileth PA Andersen J Lazarus HM Colvin OM Bennett JM Stadtmauer EA Autologous bone marrow transplant in acute myeloid leukemia in first remission. J Clin Oncol (1993) 11(2):314–9. 10.1200/JCO.1993.11.2.314 8426209

[7] Mick R Williams SF Bitran JD. Patients at increased risk for late engraftment after transplantation: a novel method for their identification. Bone Marrow Transplant (1990) 6(3):185–91. 2252958

[8] Sivakumaran M Hutchinson RM Pringle H Graham S Primrose L Wood JK Thrombocytopenia following autologous bone marrow transplantation: evidence for autoimmune aetiology and B cell clonal involvement. Bone Marrow Transplant (1995) 15(4):531–6. 7655377

[9] Sheridan WP Begley CG Juttner CA Szer J To LB Maher D Effect of peripheral-blood progenitor cells mobilised by filgrastim (G-CSF) on platelet recovery after high-dose chemotherapy. Lancet (1992) 339(8794):640–4. 10.1016/0140-6736(92)90795-5 1371817

[10] Bielski M Yomtovian R Lazarus HM Rosenthal N. Prolonged isolated thrombocytopenia after hematopoietic stem cell transplantation: morphologic correlation. Bone Marrow Transplant (1998) 22(11):1071–6. 10.1038/sj.bmt.1701499 9877269

[11] Pulanic D Lozier JN Pavletic SZ. Thrombocytopenia and hemostatic disorders in chronic graft versus host disease. Bone Marrow Transplant (2009) 44(7):393–403. 10.1038/bmt.2009.196 19684626

[12] Dominietto A Lamparelli T Raiola AM Van Lint MT Gualandi F Berisso G Transplant-related mortality and long-term graft function are significantly influenced by cell dose in patients undergoing allogeneic marrow transplantation. Blood (2002) 100(12):3930–4. 10.1182/blood-2002-01-0339 12393584

[13] Anasetti C Rybka W Sullivan KM Banaji M Slichter SJ. Graft-v-host disease is associated with autoimmune-like thrombocytopenia. Blood (1989) 73(4):1054–8. 2920206

[14] Akpek G Lee SJ Flowers ME Pavletic SZ Arora M Lee S Performance of a new clinical grading system for chronic graft-versus-host disease: a multicenter study. Blood (2003) 102(3):802–9. 10.1182/blood-2002-10-3141 12714524

[15] Bolwell B Pohlman B Sobecks R Andresen S Brown S Rybicki L Prognostic importance of the platelet count 100 days post allogeneic bone marrow transplant. Bone Marrow Transplant (2004) 33(4):419–23. 10.1038/sj.bmt.1704330 14688814

[16] Sakamaki S Hirayama Y Matsunaga T Kuroda H Kusakabe T Akiyama T Transforming growth factor-beta1 (TGF-beta1) induces thrombopoietin from bone marrow stromal cells, which stimulates the expression of TGF-beta receptor on megakaryocytes and, in turn, renders them susceptible to suppression by TGF-beta itself with high specificity. Blood (1999) 94(6):1961–70. 10477725

[17] Chatterjee S Dutta RK Basak P Das P Das M Pereira JA Alteration in marrow stromal microenvironment and apoptosis mechanisms involved in aplastic anemia: an animal model to study the possible disease pathology. Stem Cells Int (2010) 2010:932354. 10.4061/2010/932354 21048856 PMC2963319

[18] in’t Anker PS Noort WA Kruisselbrink AB Scherjon SA Beekhuizen W Willemze R Nonexpanded primary lung and bone marrow-derived mesenchymal cells promote the engraftment of umbilical cord blood-derived CD34(+) cells in NOD/SCID mice. Exp Hematol (2003) 31(10):881–9. 10.1016/S0301-472X(03)00202-9 14550803

[19] Noort WA Kruisselbrink AB in’t Anker PS Kruger M van Bezooijen RL de Paus RA Mesenchymal stem cells promote engraftment of human umbilical cord blood-derived CD34(+) cells in NOD/SCID mice. Exp Hematol (2002) 30(8):870–8. 10.1016/S0301-472X(02)00820-2 12160838

[20] Metheny L 3rd Eid S Lingas K Reese J Meyerson H Tong A Intra-osseous co-transplantation of CD34-selected umbilical cord blood and mesenchymal stromal cells. Hematol Med Oncol (2016) 1(1):41–5. 10.15761/HMO.1000105 27882356 PMC5117423

[21] da Silva Meirelles L Caplan AI Nardi NB. In search of the in vivo identity of mesenchymal stem cells. Stem Cells (2008) 26(9):2287–99. 10.1634/stemcells.2007-1122 18566331

[22] Crisan M Yap S Casteilla L Chen CW Corselli M Park TS A perivascular origin for mesenchymal stem cells in multiple human organs. Cell Stem Cell (2008) 3(3):301–13. 10.1016/j.stem.2008.07.003 18786417

[23] Caplan AI. Adult mesenchymal stem cells: when, where, and how. Stem Cells Int (2015) 2015:628767. 10.1155/2015/628767 26273305 PMC4529977

[24] Ringden O Mesenchymal stromal cells as first-line treatment of graft failure after hematopoietic stem cell transplantation. Stem Cells Dev (2009) 18(9):1243–6. 10.1089/scd.2009.1809.edi 19905962

[25] Lazarus HM Koc ON Devine SM Curtin P Maziarz RT Holland HK Cotransplantation of HLA-identical sibling culture-expanded mesenchymal stem cells and hematopoietic stem cells in hematologic malignancy patients. Biol Blood Marrow Transplant (2005) 11(5):389–98. 10.1016/j.bbmt.2005.02.001 15846293

[26] Koç ON Gerson SL Cooper BW Dyhouse SM Haynesworth SE Caplan AI Rapid hematopoietic recovery after coinfusion of autologous-blood stem cells and culture-expanded marrow mesenchymal stem cells in advanced breast cancer patients receiving high-dose chemotherapy. J Clin Oncol (2000) 18(2):307–16. 10.1200/JCO.2000.18.2.307 10637244

[27] Fouillard L Bensidhoum M Bories D Bonte H Lopez M Moseley AM Engraftment of allogeneic mesenchymal stem cells in the bone marrow of a patient with severe idiopathic aplastic anemia improves stroma. Leukemia (2003) 17(2):474–6. 10.1038/sj.leu.2402786 12592355

[28] Carrancio S Romo C Ramos T Lopez-Holgado N Muntion S Prins HJ Effects of MSC coadministration and route of delivery on cord blood hematopoietic stem cell engraftment. Cell Transplant (2013) 22(7):1171–83. 10.3727/096368912X657431 23031585

[29] Li ZY Wang CQ Lu G Pan XY Xu KL. Effects of bone marrow mesenchymal stem cells on hematopoietic recovery and acute graft-versus-host disease in murine allogeneic umbilical cord blood transplantation model. Cell Biochem Biophys (2014) 70(1):115–22. 10.1007/s12013-014-9866-y 24696072

[30] Pontikoglou C Deschaseaux F Sensebe L Papadaki HA. Bone marrow mesenchymal stem cells: biological properties and their role in hematopoiesis and hematopoietic stem cell transplantation. Stem Cell Rev (2011) 7(3):569–89. 10.1007/s12015-011-9228-8 21249477

[31] Minteer D Marra KG Rubin JP. Adipose-derived mesenchymal stem cells: biology and potential applications. Adv Biochem Eng Biotechnol (2013) 129:59–71. 10.1007/10_2012_146 22825719

[32] Parolini O Alviano F Bagnara GP Bilic G Bühring HJ Evangelista M Concise review: isolation and characterization of cells from human term placenta: outcome of the first international Workshop on Placenta Derived Stem Cells. Stem Cells (2008) 26(2):300–11. 10.1634/stemcells.2007-0594 17975221

[33] Gaberman E Pinzur L Levdansky L Tsirlin M Netzer N Aberman Z Mitigation of lethal radiation syndrome in mice by intramuscular injection of 3D cultured adherent human placental stromal cells. PLoS One (2013) 8(6):e66549. 10.1371/journal.pone.0066549 23823334 PMC3688917

[34] Prather WR Toren A Meiron M. Placental-derived and expanded mesenchymal stromal cells (PLX-I) to enhance the engraftment of hematopoietic stem cells derived from umbilical cord blood. Expert Opin Biol Ther (2008) 8(8):1241–50. 10.1517/14712598.8.8.1241 18613774

[35] McDermott SP Eppert K Lechman ER Doedens M Dick JE. Comparison of human cord blood engraftment between immunocompromised mouse strains. Blood (2010) 116(2):193–200. 10.1182/blood-2010-02-271841 20404133

[36] Ofir RP Pinzur L Levent A Aberman Z Gorodetsky R Volk HD Mechanism of action of PLX-R18, a placental-derived cellular therapy for the treatment of radiation-induced bone marrow failure. Blood (2015) 126(23):2417.

[37] Burger OA Ashtamker GF Bercovich N Rusanovsky M Pinzur L Jacov OM Human placental derived mesenchymal stromal cells (MSC) grown in 3D-culture (PLX-I), promotes engraftment of human umbilical cord blood (hUCB) derived CD34+ cells in NOD/SCID mice. Blood (2007) 110(11):1416.

[38] Hiwase SD Dyson PG To LB Lewis ID. Cotransplantation of placental mesenchymal stromal cells enhances single and double cord blood engraftment in nonobese diabetic/severe combined immune deficient mice. Stem Cells (2009) 27(9):2293–300. 10.1002/stem.157 19544531

[39] Allen HS Sher N Rekhes S Pinzur L Prezma T Gorodetsky R Human placenta-derived stromal cells rescue mice from radiation-induced bone marrow failure: a Cytof-based mechanistic analysis. Blood (2016) 128(22):2677.

[40] Or RG Grisaro S Avni BR Resnick I Dari L Shoshani D Correction of post-transplant hematopoiesis by novel use of mesenchymal-like placental expanded cells (PLX) administered intra-muscular. Blood (2012) 120(21):4133.

